# Engineered fungal polyketide biosynthesis in *Pichia pastoris*: a potential excellent host for polyketide production

**DOI:** 10.1186/1475-2859-12-77

**Published:** 2013-09-08

**Authors:** Limei Gao, Menghao Cai, Wei Shen, Siwei Xiao, Xiangshan Zhou, Yuanxing Zhang

**Affiliations:** 1 State Key Laboratory of Bioreactor Engineering, East China University of Science and Technology, Shanghai 200237, China

**Keywords:** Polyketide, *Pichia pastoris*, 6-MSA, 6-MSAS, Heterologous expression, Fermentation

## Abstract

**Background:**

Polyketides are one of the most important classes of secondary metabolites and usually make good drugs. Currently, heterologous production of fungal polyketides for developing a high potential industrial application system with high production capacity and pharmacutical feasibility was still at its infancy. *Pichia pastoris* is a highly successful system for the high production of a variety of heterologous proteins. In this work, we aim to develop a *P. pastoris* based *in vivo* fungal polyketide production system for first time and evaluate its feasibility for future industrial application.

**Results:**

A recombinant *P. pastoris* GS115-NpgA-ATX with *Aspergillus nidulans* phosphopantetheinyl transferase (PPtase) gene *npgA* and *Aspergillus terrus* 6-methylsalicylic acid (6-MSA) synthase (6-MSAS) gene *atX* was constructed. A specific compound was isolated and idenified as 6-MSA by HPLC, LC-MS and NMR. Transcription of both genes were detected. In 5-L bioreactor, the GS115-NpgA-ATX grew well and produced 6-MSA quickly until reached a high value of 2.2 g/L by methanol induction for 20 hours. Thereafter, the cells turned to death ascribing to high concentration of antimicrobial 6-MSA. The distribution of 6-MSA changed that during early and late induction phase it existed more in supernatant while during intermediate stage it mainly located intracellular. Different from 6-MSA production strain, recombinant *M. purpureus pksCT* expression strains for citrinin intermediate production, no matter PksCT located in cytoplasm or in peroxisomes, did not produce any specfic compound. However, both *npgA* and *pksCT* transcripted effectively in cells and western blot analysis proved the expression of PPtase. Then the PPTase was expressed and purified, marked by fluorescent probes, and reacted with purified ACP domain and its mutant ACPm of PksCT. Fluoresence was only observed in ACP but not ACPm, indicating that the PPTase worked well with ACP to make it bioactive *holo*-ACP. Thus, some other factors may affect polyketide synthesis that include activities of the individual catalytic domains and release of the product from the synthase of PksCT.

**Conclusions:**

An efficient *P. pastoris* expression system of fungal polyketides was successfully constructed. It produced a high production of 6-MSA and holds potential for future industrial application of 6-MSA and other fungal polyketides.

## Background

Polyketides are one of the most important classes of secondary metabolites. They occur in bacteria, fungi, plants, marine organisms, etc., showing flexible structural diversity and a variety of bioactivities [1]. Polyketides and their derivatives make good drugs. As reported by Weissman and Leadlay in 2005, polyketide-derived pharmaceuticals comprise 20% of the top-selling drugs, with combined worldwide revenues of over UK £10 billion per year [2]. Many important drugs have suffered or will face expiration of the key patents, which is likely to lead to explosive production growth of these compounds [3].

During the last two decades, the biosynthetic mechanism of polyketides catalyzed by polyketide synthase (PKS) have been well interpreted despite that some highly controlled synthetic processes still remained unknown. As is known, fungal PKSs belong to iterative type I PKS that contains an obligatory set of ketosynthase (KS), acyltransferase (AT) and acyl carrier protein (ACP) domains, which is closely related to fatty acid biosynthesis [1].The ACP domains carry the malonyl extender units and transiently link the growing acyl chain. Before functioning, the *apo*-ACP requires post-translational modification to be *holo*-ACP through the addition of phosphopantetheine (PP) derived from coenzyme A (CoA) to a conserved serine residue catalyzed by phosphopantetheinyl transferase (PPtase). The Claisen condensation is catalyzed by the KS while the AT transfers acyl groups from CoA onto the KS and ACP domains. The resulted β-ketothioester could be further processed by β-ketoacyl reductase (KR), dehydratase (DH) and/or enoyl reductase (ER) to form an ester with different degree of saturation. Fungal PKS can own another intrinsic domain C-methyl transferase (CMeT) that additionally methylated the chain using a methyl group from S-adenosylmethionine (SAM). Once the polyketide chain has finished, a thioesterase (TE) hydrolyses the thioester and releases the carbon chain from the enzyme, although it is not found in every PKS. The recycling mechanism of fungal type I PKS makes the biosynthesis process efficiently with relatively small genes.

Due to the broad bioactivity and well known synthesis mechanism of polyketides their production have attracted much interest in the field of metabolic engineering. To present, several hosts, i.e., *E.coli*, *Saccharomyces cerevisiae*, *Streptomyces coelicolor*, *Aspergillus nidulans*, *Aspergillus oryzae*, etc., have already been accomplished for polyketides biosynthesis [4]. An reconstruction of a complete functional fungal biosynthetic multigene cluster even succeeded in *A. oryzae*[5]. However, these studies mainly focused on identifying the functions of PKSs, performing combinational biochemistry for new compound biosynthesis, or testing the feasibility of the expression system, while the work for developing a high potential industrial application system with high production capacity of pharmaceutical compound was still at its infancy. It is believed that, combinational biochemistry would play an important role in future drug development and explore a highly efficient heterologous production system turns to be critical.

As is known, *Pichia pastoris* is well used as a highly successful system for the production of a variety of heterologous proteins in both laboratory and industry [6,7]. For *P. pastoris*, the simple molecular genetic manipulation and commercially available kit made it conveniently for laboratory and industrial application. It can produce foreign proteins at high levels under control of methanol-inducible strong promoter *AOX1*, either intracellularly or extracellularly. Moreover, it has the capability of directing proteins into peroxisome and performing many eukaryotic post-translational modifications, such as glycosylation, disulfide bond formation and proteolytic processing. However, till now, there is few reports focused on polyketide synthase (PKS) expression triggering compound biosynthesis *in vivo* by *P. pastoris*.

The PKS of 6-methylsalicylic acid (6-MSA) synthase (6-MSAS) responsible for 6-MSA biosynthesis was the first fungal PKS gene to be cloned and is also one of the best characterized fungal PKSs [8-10]. It has been commonly used as a model PKS for evaluation of heterologously expression system such as *S. cerevisiae*[3,11],*S. coelicolo*r [12], *E. coli*[13], tobacco [14]. Mycotoxin citrinin was originally isolated from *Penicillium citrinum* and now produced by a variety of other fungi [15]. It damages human health and usually accompanies with pigment production in *Monascus* spp., and gives rise to wide attention. A gene cluster for biosynthesis of mycotoxin citrinin have been reported, in which, a *pksCT* plays an important role and probably responsible for its intermediate biosynthesis [16,17]. For heterologous production of polyketide in yeast, the post-translational modification of the ACP by phosphopantetheinylation is necessary. As previously reported, this challenge could be solved by the PPTase encoding genes of *npgA* from *A. nidulans* or *sfp* from *Bacillus subtilis*[3,13].

In this work, we dedicated to establish a *P. pastoris* based polyketide production system. The synthetic pathway of model fungal polyketide 6-methylsalicylic acid encoded by *atX* from *Aspergillus terreus*[10,18] and citrinin intermediate encoded by *pksCT* from *Monascus purpureus* were constructed to verify the feasibility of heterologous production of fungal polyketide in *P. pastoris*. Besides, bioreactor fermentation was also performed to evaluate the polyketide productivity of the recombinant strain*.*

## Results and discussion

### Construction of strain GS115-NpgA, GS115-ATX and GS115-NpgA-ATX

For polyketide biosynthesis, the ACP functions after post-translational modification by phosphopantetheine transferase (PPTase), leading to the transfer of 4′-phosphopantetheine from coenzyme A (CoA) to a conserved serine residue of ACP [1]. In this study, *A. nidulans npgA* (GenBank: AAF12814) encoding the PPTase consisted of 344 amino acids was selected. Strains were constructed following Section Plasmids and strains. Commercial vector pPIC3.5 K (Invitrogen) with *AOX1* promoter and selective marker *HIS4* was used for plasmid pPIC3.5 K-NpgA construction and then transformed auxotrophic *his4* wild type *P. pastoris* GS115 by a single homologous recombination event to integrate at *HIS4* locus. A positive transformant *P. pastoris* GS115-NpgA was screened and fully identified by PCR. The 6-MSAS containing 1803 amino acids encoded by *atX* (GenBank: D85860) from *A. terrus* was expressed by *P. pastoris* and purified with Nickel-affinity chromatography. The intron removed *atX* was cloned from a gift plasmid pESC-ATX. The vector pPICZ B (Invitrogen) with *AOX1* promoter and selective marker of zeocin resistance gene *Sh ble* was used for plasmid pPICZ B-ATX construction and then transformed *P. pastoris* GS115 and recombinant *P. pastoris* GS115-NpgA by single homologous recombination events to integrate at 5′*AOX1* region. The positive methanol utilization plus (Mut^+^) transformants GS115-ATX and GS115-NpgA-ATX were screened and fully identified by PCR analysis.

### Product identification of GS115-NpgA-ATX

**Figure 1:**
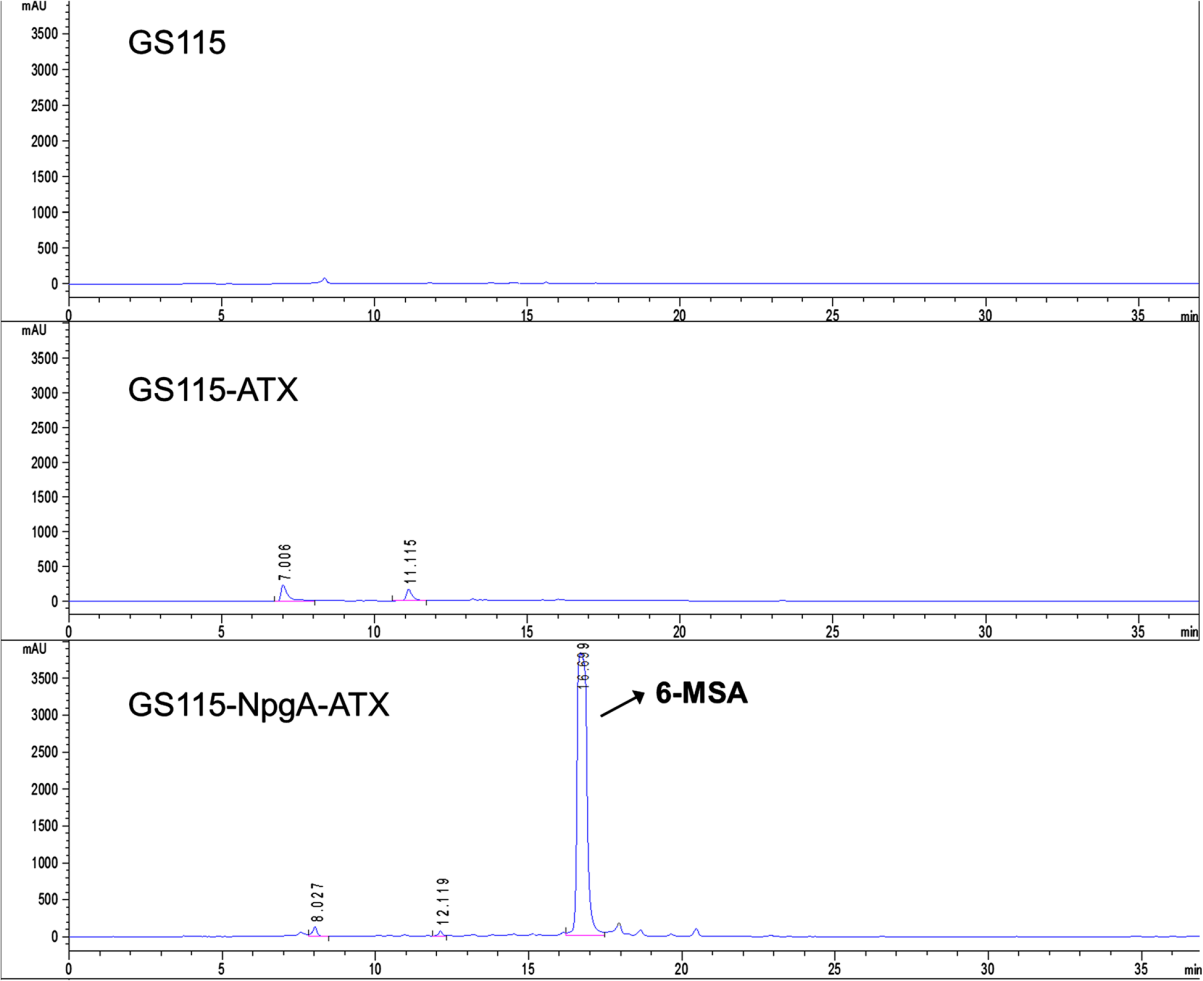
**The HPLC chromatogram of organic extracts from fermentation broth of strain GS115, GS115-ATX and GS115-NpgA-ATX.** Samples preparation were described in the Section 6-MSA extraction and identification in Methods.

**Figure 2:**
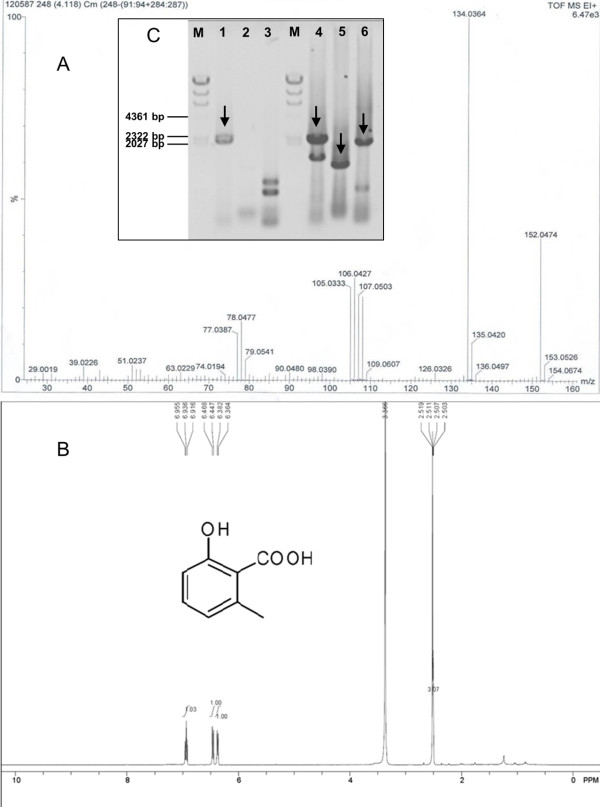
**The EI-MS identification of product 6-MSA by GS115-NpgA-ATX and transcription analysis of *npgA *and *atX*. (A)** EI-MS analysis of extract from GS115-NpgA-ATX. m/z, mass-to-charge ratio; **(B)** The ^1^HNMR analysis of extract from GS115-NpgA-ATX. Samples preparation were in the Section 6-MSA extraction and identification in Methods; **(C)** Lane 1–3: PCR using cDNA of wild type GS115 and primers 5AOX1/3AOX1, NpgA-F/R1 and BstpF/AtxR, respectively; Lane 4–6: PCR using GS115-NpgA-ATX cDNA and primers 5AOX1/3AOX1, NpgAF/ NpgAR1 and BstpF/AtxR, respectively; M: DNA marker Hind III. Gene transcription induced by 0.5% methanol for 24 h.

Three strains, GS115, GS115-ATX and GS115-NpgA-ATX, were cultivated under same conditions. After methanol induced expression for 36 h, the products were extracted and analyzed by HPLC analysis. As shown in Figure 1, GS115-NpgA-ATX produced a specfic compound emerged as a sharp peak in HPLC chromatogram at 16.7 min compared with GS115 and GS115-ATX, which had the same retention time as an authentic sample of 6-MSA under this elution condition. To further confirm the structure of the compound, the extracts were purified by TLC (petroleum ether:ethyl acetate = 3:1 (v/v), 1% acetic acid) and extracted and freeze-dried for further EI-MS and NMR analysis. The EI-MS analysis was performed on an Agilent G2577A mass spectrometer, establishing the molecular formulae as C_8_H_8_O_3_ for 6-MSA (with the M^+^ ion at m/z = 152.0474, 152.0473 calculated) absolutely accorded with the standard 6-MSA (Figure 2A). The freeze-dried sample dissolved in deuterated DMSO for ^1^HNMR analysis. The results that ^1^HNMR (400 MHz, DMSO-d6),δH = 6.37 (1H, m, H-3), 6.94 (1H, m, H-4), 6.46 (1H, m, H-5), 2.52 (3H, s, CH_3_-6) conformed with ^1^HNMR spectrum of standard 6-MSA and further confirmed the compound (Figure 2B). For all three tested strains, only GS115-NpgA-ATX produced 6-MSA but not GS115-ATX and wild type GS115, proving that 6-MSAS could be well modified by PPtase and it only works for 6-MSA biosynthesis after PPtase modification. Furthermore, transcription analysis of GS115 and GS115-NpgA-ATX were carried out after 24 hours induction. Three pairs of primers, 5AOX1/3AOX1, NpgAF/NpgAR1, BstpF/AtxR, were used to test gene transcriptions of *AOX1*, *npgA* and *atX*. The wild type GS115 only generated the correct nucleic acid size of 2.2 kb for the *AOX1*, while GS115-NpgA-ATX formed other two desired PCR products (1.3 kb for *npgA* and 1.9 kb for *atX*) besides the *AOX1* (Figure 2C), indicating that both *npgA* and *atX* transcripted effectively in the recombinant strain GS115-NpgA-ATX.

### Fermentation of GS115-NpgA-ATX in 5-L bioreactor

**Figure 3:**
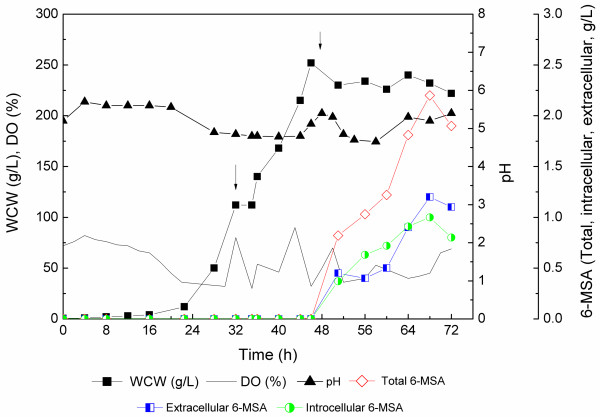
**Time profiles of GS115-NpgA-ATX in 5-L stirred-tank bioreactor fermentation.** The first arrow (32 h) indicated the starting point of glycerol feeding; The second arrow (47 h) indicated the starting point of methanol induction. Culture conditions were shown in Methods.

A 5-L bioreactor fermentation was subsequently conducted to evaluate the productivity of 6-MSA by the recombinant GS115-NpgA-ATX. As shown in Figure 3, a batch phase in BSM medium proceeded until 32 h when glycerol used up accompanied with dissolved oxygen sharply increase, and reached a wet cell weight (WCW) of 112 g/L. A fed-batch phase continued with limited feedings of glycerol feeding medium to improve cell growth until 46 h when a high cell density of 252 g/L (WCW) was achieved. Then, methanol induction phase started at 47 h after glycerol exhaused with derepression of *AOX1* promoter. The 6-MSA rapidly synthesized and accumulated. After induction for 20 hours, 6-MSA production reached up to the highest 2.2 g/L. However, the culture broth turned extremely abnormal and cells turned to death by methylene blue staining analysis. During methanol induction phase, cell growth was highly repressed, which should be ascribed to the antimicrobial activity of 6-MSA [19]. The repression effect of 6-MSA on *P. pastoris* growth was subsequently verified in 5-L bioreactor fermentation with commerially purchased 6-MSA addition with final concentration of 2.05 g/L immediately after WCW reached 235 g/L (Additional file 1: Figure S1). For GS115-NpgA-ATX, the distribution of 6-MSA changed that during early induction phase and late phase it existed more in supernatant while during intermediate stage it mainly located intracellular. It might attribute to that the increased methanol feeding rate up-regulated the expression rate of 6-MSAS so that enhanced 6-MSA synthesis. The rapidly accumulated 6-MSA could not be efficiently transferred extracellular. However, during the late induction phase, the death of cells may accelarated 6-MSA release to fermentation broth.

Kealey et al. reported a heterogous expression of 6-MSA with a low production of 75 mg/L in *E. coli* and a high production of 1.7 g/L in *S. cerevisiae* with coexpression of *Penicillium patulum* MSAS and *B. subtilis* SFP [13]. Wattanachaisaereekul et al. obtained a 6-MSA production of 0.2 g/L by heterologous expression of *A. nidulans* NpgA and *B. subtilis* SFP [3]. They then optimized the expression by swapping promoter *P*_*ACC1*_ to a strong, constitutive promoter *P*_*TEP1*_ to increase malonyl-CoA supply and resulted in an increased 6-MSA production of 0.5 g/L [20]. The 6-MSA of 2.2 g/L in this case is higher than these published data. It could be further to go by promoting cell density before methanol inducation or developing a fermentation and bioseparation coupling process that releasing 6-MSA from broth to keep cell alive.

### Product, transcription and western blot analysis of PksCT expression strains

The previous study showed that a 56 bp intron presented at 640 to 695 bp, flanked by a typical splice site (5′-GT-AG-3′) in *pksCT*, a gene critical for citrinin biosynthesis in *M. purpureus*[21]. The PksCT possessed KS-AT-ACP-MT domains but no TE domain [21]. However, in this study, we found another intron located at 6547 to 6608 bp beside the reported one by RT-PCR. The cDNA of *pksCT* by removing two introns were 6507 bp, coded for 2169 amino acids, which also contained the intact KS-AT-ACP-MT domains. The expression strains, i.e., GS115-3.5 K, GS115-3.5 K-CT-SKL, GS115-NpgA-CT-SKL, GS115-NpgA-SKL-CT-SKL and GS115-NpgA-CT, were then constructed as that described in Methods and the integration mechanism were the same to construction of *atX* recombinant strains. However, different from 6-MSAS expression strains, none of them produced specific compound comparing with the control GS115 by HPLC-MS. The strains expressing *pksCT* that only removed the front 56 bp intron were also constructed but were still nothing different (data not shown).

**Figure 4:**
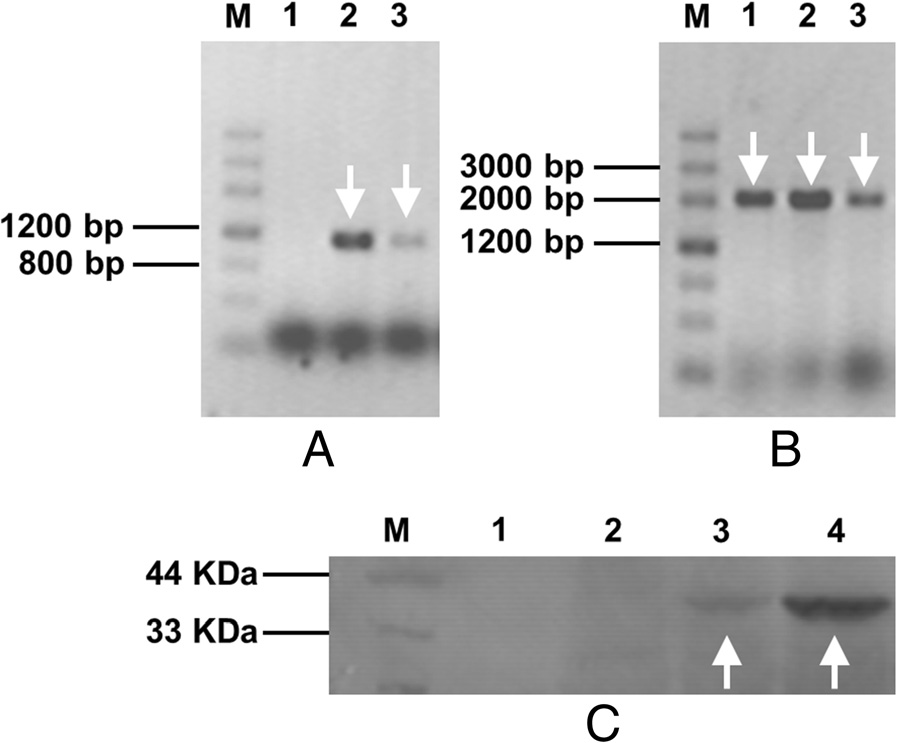
**Transcription of *npgA *and *pksCT *and western blot assay of PPtase induced by 0.5% methanol. (A)** PCR with primers NpgA-HIS6-F/ NpgA-HIS6-R; **(B)** PCR with primers 1977 F/4088R. Lane 1: GS115-3.5 K-CT-SKL; Lane 2: GS115-NpgA-CT; Lane 3: GS115-NpgA-SKL-CT-SKL. **(C)** Western blot of PPtase. Lane1: GS115; Lane2: GS115-3.5 K-CT-SKL; Lane 3: GS115-NpgA-CT; Lane 4: GS115-NpgA-SKL-CT-SKL. The arrows indicates the positive bands of nucleic acids and proteins. Samples preparation and experimenal procedure were shown in the Section Transcription and western blot analysis in Methods.

Transcription of GS115-3.5 K-CT-SKL, GS115-NpgA-CT and GS115-NpgA-SKL-CT-SKL were then analyzed after 24 hours induction. Two pairs of primers, NpgA-HIS6-F/ NpgA-HIS6-R and 1977 F/4088R were used to test gene transcriptions of *npgA* and *pksCT*. GS115-3.5 K-CT-SKL acted no *npgA* transcription but GS115-NpgA-CT and GS115-NpgA-SKL-CT-SKL did (1.1 kb) (Figure 4A). As for *pksCT*, all three strains transcripted normally (2.1 kb) (Figure 4B). A western blot analysis detecting PPtase expression was further applied (Figure 4C). The GS115-NpgA-CT and GS115-NpgA-SKL-CT-SKL clearly presented the PPtase band of about 40 KDa while neither did GS115 nor GS115-3.5 K-CT-SKL. These results suggested that genes transcription and PPtase expression functioned well.

### Phosphopantetheinylation activity analysis of PPTase on ACP domain of PksCT

**Figure 5:**
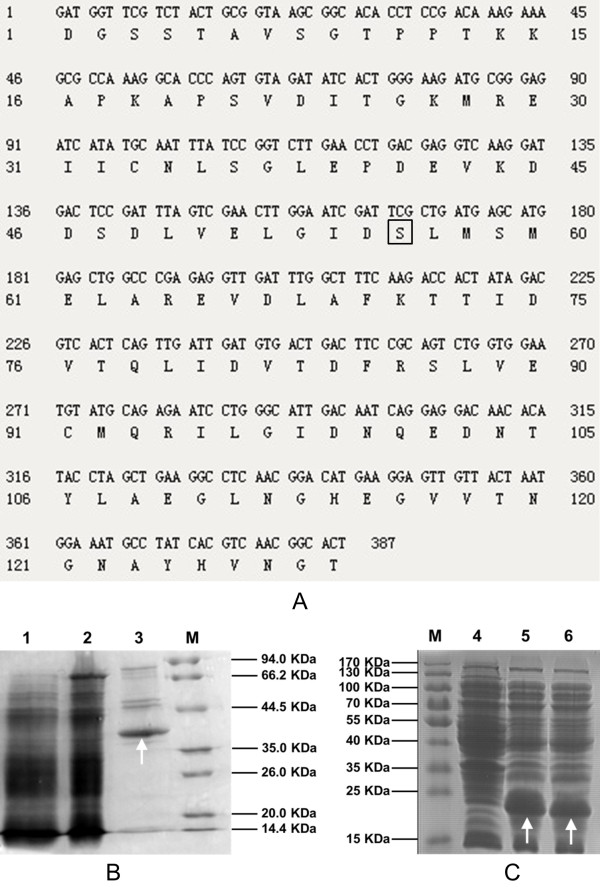
**Nucleotide and amino acid sequences of ACP amd SDS-PAGE of PPTase and ACP and ACPm. (A)** nucleotide and amino acid sequences ACP domain of citrinin polyketide synthase. The conserved serine at site 56 which marked by square frame was mutated by overlap PCR using primers mutant5/mutant3 to generate ACPm. **(B)** SDS-PAGE of PPTase expressed by GS115-NpgA-HIS_6_; **(C)** SDS-PAGE of ACP and ACPm expressed by *E. coli* BL21. Lane 1: Lysate supernatant of GS115-NpgA-HIS_6_; Lane 2: Flow-through fraction of GS115-NpgA-HIS_6_; Lane 3: Eluted protein of GS115-NpgA-HIS_6_; Lane 4: Lysate supernatant of wild type *E. coli* BL21 strain as negative control; Lane 5: Lysate supernatant of BL21-ACP; Lane 6: Lysate supernatant of BL21-ACPm. M: Protein marker. Protein purification were described in the Section Protein expression and purification in Methods. The arrows indicates the objective proteins.

To determine whether or not *A. nidulans* PPtase could react with ACP domain of *M. purpureus* PksCT, an *in vitro* experiment was then studied. The PPTase containing 344 amino acids encoded by *A. nidulans npgA* (GenBank: AAF12814) was expressed by *P. pastoris* and purified with Nickel-affinity chromatography. The molecular weight (Mw) of PPtase with His taq was calculated as 40 KDa by the software Scansite pI/Mw. As shown in Figure 5B, PPTase clearly exhibited in SDS-PAGE with a Mw about 40 Kda.

The ACP domain of fugnal PKS citrinin synthase (PksCT) from *M. purpureus* was employed to test the activity of PPtase. The ACP domain was analyzed and determined by Udwary–Merski algorithm (UMA) procedure (Figure 5A) [22,23] relying on the assumptions that amino acids in domain regions are relatively more conserved while linker regions carry more mutations and higher hydrophilicity [24]. *E. coli* BL21 was used for expression of ACP and its mutant ACPm (conserved serine at site 56 to alanine) (Figure 5A), and the proteins clearly appeared in the SDS-PAGE with similar Mw of about 20 Kda (Figure 5C).

The bioactivity of PPtase was evaluated based on fluorescence labelled strategy. As we know, for function of PKS, the *apo*-ACP requires post-translational modification to form *holo*-ACP through the addition of phosphopantetheine derived from coenzyme A (CoA) to a conserved serine residue catalyzed by PPtase. If the conserved serine is mutated, the phosphopantetheinylation will then fail. Fluorescent probes Alexa Fluor 647-C2maleimide and Bodipy FL N-(2-aminoethyl)maleimide can label phosphopantetheine by reacting with its hydrosulphonyl group. When fluorescently labelled 4′-phosphopantetheine linked with *apo*-ACP, the resulted *holo*-ACP would show fluorescence and stabilize in the SDS-PAGE [25]. However, once the conserved serine of ACP was mutated, it could not be labelled and emit fluorescence. In point of this study, once the PPtase encoded by *A. nidulans npgA* works, Alexa Fluor-647-phosphopantetheine labelled ACP would emit red fluorescence with 480 nm exciting light and 535 nm filter, while Bodipy FL-phosphopantetheine labelled ACP would emit green fluorescence with 580 nm exciting light and 670 nm filter.

**Figure 6:**
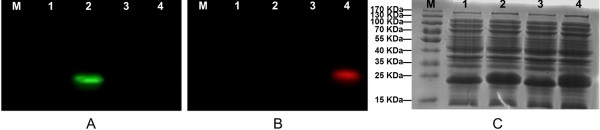
**Fluorescent assay of ACP and ACPm that were fluorescently labelled.** The KODAK In-vivo multispectral imaging systerm F was used for scanning. **(A)** Image parameter of 480 nm laser and 535 nm emission filter; **(B)** Image parameter of 580 nm laser and 670 nm emission filter; **(C)** Image of the same SDS-PAGE gel stained with Coomassie brilliant blue R-250. Lane 1: *In vitro* reaction containing Bodipy FL-CoA, PPtase and ACPm; Lane 2: *In vitro* reaction containing Bodipy-CoA, PPtase and ACP; Lane 3: *In vitro* reaction containing Alexa Fluor 647-CoA, PPtase and ACPm; Lane 4: *In vitro* reaction containing Alexa Fluor 647-CoA, PPtase and ACP. Protein preparation were described in Methods.

As shown in Figure 6, Lane 1 and 3 were loaded with ACPm (conserved serine at site 56 to alanine) of PksCT that deactivating transthioesterification while Lane 2 and 4 contained the correct ACP of PksCT without mutation. Bodipy FL- phosphopantetheine labelled ACP in Lane 2 emitted green fluorescence when excited with wavelength of 480 nm (Figure 6A), while Alexa Fluor-647- phosphopantetheine labelled ACP in Lane 4 produced red fluorescence when excited with wavelength of 580 nm (Figure 6B). However, Lane 1 and 3 emitted no fluorescence under different wavelengths of exciting lights and filters (Figure 6A&B). The gel was then stained by Coomassie brilliant blue R250 solution and both ACP and ACPm presented correct Mw (Figure 6C). The results proved that *A. nidulans* PPtase possessed good bioactivity for catalyzing modification of *M. purpureus* ACP. However, if the modification sites were mutated, i.e., ACPm, it lost ability of phosphopantetheinylation so that deprived transthioesterification function for polyketide biosynthesis. This indicated that inefficient phosphopantetheinylation should not be the reason why citrinin intermediate was not observed. Other factors may affect polyketide synthesis that include activities of the individual catalytic domains and release of the product from the synthase [25]. The pksCT may also be poorly active without other proteins. Cox and coworkers reported that coexpression of different genes from fungal PKS clusters could lead to increased titres of objective compounds [5,26]. Coexpression of *tenB* with *tenA*, *tenC* and *tenS* with individual promoters and terminators increased Pretenellin-B by 149.2% in heterogolous expression in *A. oryzae* as compared to that without *tenB*[5]. Also, a hybrid fungal PKS-NRPS system TENS-PKS:DMBS-NRPS appeared to be more active than either of its progenitors and produced much higher purified yields of prototenellin C [26]. Additionally, some correct fungal polyketides may only be obtained by coexpression of their encoded PKS genes and other related genes. For instance, expression of *tenS* in the absence of a trans-acting ER component encoded by *orf3* led to errors in assembly of the polyketide component, while coexpression of both genes worked correctly [27]. The *lovF* encoding lovastatin nonaketide synthase only functioned accurately with coexpression with *lovC* encoding a trans-acting enoyl reductase in the heterologous production of monacolin-J [28]. Sakai et al. have recently reported a successful case of heterologous production of citrinin in *A. oryzae* by involving a small citrinin gene cluster (less than 20 kb) containing *pksCT*, activator gene *ctnA* and other 4 ORFs [16]. It is necessary for us to express this gene cluster or coexpression *pksCT* with related genes to further evaluate the *P. pastoris* system and probe into the source for this unsuccessful expression of *pksCT* gene.

Previous study have succeeded in heterologous production of fungal polyketides by involving *A. nidulans* PPtase, revealing that PPtase worked well on ACP of 6-MSAS and LNKS origining from *P. patulum* and *A. terru*s, respectively [13,25]. In this study, *A. nidulans* PPtase reacting with ACP of *M. purpureus* was also proven. These results indicated that *A. nidulans* PPtase probably work on ACPs from a wide range of filamentous fungi.

This work revealed that *P. pastoris* could be a good host that serve for heterogous expression of native polyketide or engineering recombinant polyketide biosynthesis in lab study. It also held great potential for heterologous production of polyketides in industrial application. We believed that *P. pastoris* could show more strong productive ability in heterologous production of non-lethal polyketides. Moreover, in view of the relative ease of performing genetic engineering the metabolic network modification may readily acheived to enhance precursor or intermediate formation or weaken its competing pathway for polyketide biosynthesis in *P. pastoris*. A successful case expanding the range of starter and extender units for polyketide biosynthesis has been illustrated in *S. cerevisiae* by introducing pathways for methylmalonyl-CoA generation [11]. However, the *P. pastoris* system may also faces the chellenges that other hosts ever encountered. The presence of introns and strain-specific splicing mechanisms for fungal PKS genes may bring hardness in verification of full length cDNA or cumbersome removal of introns [4].The huge size of gene cluster encoding the biosynthetic pathway of polyketides may render expression constructs instable [4]. Also, some unknown origins caused blocks of compound synthesis, such as citrinin intermediate in this study and lovastatin precursors in *S. cerevisiae*[25]. Anyway, *P. pastoris* showed great potential to be a good host for polyketide production, and applying *P. pastoris* for a full PKS gene cluster for compound production would deeply test out its prospect in industrial application and becomes another research key point in the near future.

## Conclusions

An efficient *P. pastoris* expression system of fungal polyketides was constructed. The recombinant *atX* strain successfully synthesized the objective compound 6-MSA and produced a high concentration (2.2 g/L) of 6-MSA in 5-L bioreactor fermentation. The *P. pastoris* expression system holds good potential for future industrial application of 6-MSA and other fungal polyketides, despite that its universality and the mystery of ineffectual cases still need further research.

## Methods

### Molecular biology techniques

Oligonucleotides were synthesized by Shanghai Generay Biotech Co., Ltd., China (Table 1). For PCR experiments, standard protocols were applied following PCR amplification kit (TaKaRa, cat. no. R011). Fungal genomic DNA was extracted by Plant Genomic DNA Extraction Kit (Tiangen Biotech Co., Ltd., China). *E.coli* vector pET28a was purchased from TaKaRa. *P. pastoris* GS115, *E.coli* TOP10, *E.coli* BL21 and yeast vectors pPICZ B and pPIC3.5 K were purchased from Invitrogen. *A. nidulans* A1149 (TN02A3) was bought from Fungal Genetics Stock Center, USA. Fluorescent probes Alexa Fluor 647-C2maleimide and Bodipy FL N-(2-aminoethyl)maleimide were purchased from Invitrogen. DNase I was bought from Promega. Transformation of yeast cells and transformants screening were carried out according to *Pichia protocols* by Cregg and Russell [29]. Transformation and other standard recombinant DNA operations used in this study for *E. coli* were performed as described previously [30].

**Table 1:** Primers used in this study

**Primer**	**Sequence (5′ to 3′)**^**a**^
NpgA-HIS6-F	GGACCGCGGCCACCATGGTGGACTACAAGGATGACGATGACAAGGGT
	GGAGGTGGATCTGTGCAAGACACATCAAGCGCAAG
NpgA-HIS6-R	ATAAGAATGCGGCCGCGCGGATAGGCAATTACACACC
5AOX1	GACTGGTTCCAATTGACAAGC
3AOX1	GCAAATGGCATTCTGACATCC
AcpF	CATGGCTAGCGATGGTTCGTCTACTGCGGT
AcpR	CCGCTCGAGTGCTGATTTTGGGAGGATGGAACC
Mutant5	TGGAATCGATGCGCTGATGAGCATG
Mutant3	CATGCTCATCAGCGCATCGATTCCA
NpgAF	GGAATTCTACGTAACCATGGTGCAAGACACATCAAGCGCAAG
NpgAR1	ATGGGTACAGATCCTCTTCTGAGATGAGTTTTTGTTCGGATAGGCAATTACACACCCCAGTC
NpgAR2	ATAAGAATGCGGCCGCTTAAGCGTAATCTGGAACATCGTATGGGTACAGATCCTCTTCTGAGATG
AtxF	CGGGAATTCACCATGGAGGTACATGGAGATGAAGTG
AtxR	ATAAGAATGCGGCCGCCTTTCCCATCTTTTCCAAAAACCAT
BstpF	AACTGCAGAAGAATTCGGTTACCTGTACGTGGAAAAGGCTG
BstpR	AACTGCAGAAGGTAACCCACCTTGGGAGGCGGCTG
CTAF	TCCCCGCGGCATATGCAATTTATCCGGTCTTG
CTAR	ATAAGAATGCGGCCGCTTACAACTTAGAATCTAGAAATCCCATGGTCTTCC
CTBF	TCCCCGCGGTCGCGAGTACGAAGGTTTATC
CTBR	GGAATTCCATATGATCTCCCGCATCTTC
CTCF	CGCCGCGGGACATGGCTCATCATCACCATCACCATGGTGGAGGTGGATCTATGATTGACTCAACTTCGCACTC
CTCR	CTAGCCTCGCGATCACACCAAATACC
Citrinin-F2	CCATCGATTCGCTGATGAGCATGGAG-3
Citrinin-R2	GGGAATTCGCGGCCGCATGATCTCTTGGCGCCCTG
NpgASR1	ATGGGTACAGATCCTCTTCTGAGATGAGTTTTTGTTCGGATAGGCAATTACACACCCCAGTC
NpgASR2	ATAAGAATGCGGCCGCTTACAACTTAGAAGCGTAATCTGGAACATCGTATGGGTACAGAT CCTCTTCTGAGATG
1977 F	TTCCTCAGCCCTACTGGTCAAT
4088R	GCTGGGATGCGTCTTGATAACC

^a^ Restriction sites are underlined.

### Plasmids and strains

The plasmids and strains used in this study were listed in Table 2.

**Table 2:** List of plasmids and strains in this work

**Plasmids**	**Characteristic(s)**	**Reference**
pET28a(+)	Kan^R^; T7 promoter based *E. coli* expression vector	Novagen
pET28a-ACP	pET28a(+) derivative carrying *M. purpureus* ACP encoded DNA	This study
pET28a-ACPm	pET28a(+) derivative carrying *M. purpureus* ACPm encoded DNA	This study
pESC-ATX	Amp^R^; *URA3*; *P*_*GAL1*_/_*GAL10*_-based yeast expression vector; commerial pESC-URA derivative carrying *atX* gene	Moriguchi et al., 2006
pPIC3.5 K	Amp^R^ G418^R^; *HIS4*; *P*_*AOX1*_-based yeast expression vector	Invitrogen
pPICZ B	Zeocin^R^ ; *P*_*AOX1*_-based expression yeast vector	Invitrogen
pPIC3.5 K-NpgA	pPIC3.5 K derivative carrying *A. nidulans npgA* gene	This study
pPICZ B-NpgA-HIS_6_	pPICZ B derivative carrying *A. nidulans npgA* gene	This study
pPICZ B-ATX	pPICZ B derivative carrying *A. terreus atX* gene	This study
pPICZ B-CT-SKL	pPICZ B derivative carrying *M. purpureus pksCT* gene a *P. pastoris* SKL sequence	This study
pPICZ B-CT	pPICZ B derivative carrying *M. purpureus pksCT* gene	This study
pPIC3.5 K-NpgA-SKL	pPIC3.5 K derivative carrying *A. nidulans npgA* gene and a *P. pastoris* SKL sequence	This study
**Strains**	**Characteristic(s)**	Reference
GS115	*his4*, *AOX 1*, *AOX2*	Invitrogen
GS115-NpgA-HIS_6_	Zeocin^R^; GS115 with plasmid pPICZ B-NpgA-HIS_6_	This study
GS115-ATX	Zeocin^R^; GS115 with plasmid pPICZ B-ATX	This study
GS115-NpgA	G418^R^; GS115 with plasmid pPIC3.5 K-NpgA	This study
GS115-NpgA-ATX	Zeocin^R^; G418^R^; GS115-NpgA with plasmid pPICZ B-ATX	This study
GS115-3.5 K	G418^R^; GS115 with pPIC3.5 K	This study
GS115-3.5 K-CT-SKL	Zeocin^R^; G418^R^; GS115-3.5 K with pPICZ B-CT-SKL	This study
GS115-NpgA-CT	Zeocin^R^; G418^R^; GS115-NpgA with plasmid pPICZ B-CT	This study
GS115-NpgA-SKL	G418^R^; GS115 with plasmid pPIC3.5 K-NpgA-SKL	This study
GS115-NpgA-CT-SKL	Zeocin^R^; G418^R^; GS115-NpgA with plasmid pPICZ B-CT-SKL	This study
GS115-NpgA-SKL-CT-SKL	Zeocin^R^; G418^R^; GS115-NpgA-SKL with plasmid pPICZ B-CT-SKL	This study
*E. coli* TOP10	F’ [lacIq, Tn10(TetR)] *mcr*A φ80*lac*ZΔM15 Δ*lac*X74 *deo*R *rec*A1	Invitrogen
*E. coli* BL21	F^–^*ompT hsdS*_*B*_(r_B_^–^ m_B_^–^) *gal dcm* (DE3)	Invitrogen
BL21-ACP	Kan^R^; BL21 with plasmid pET28a-ACP	This study
BL21-ACPm	Kan^R^; BL21 with plasmid pET28a-ACPm	This study

### Strain GS115-NpgA , GS115-NpgA-ATX and GS115-ATX

Using primers NpgAF/NpgAR1 to amplify *A. nidulans npgA*, the obtained product was diluted 10 times and then used as DNA template to amplify *npgA* with *C-*terminal c-myc-HA taq by primers NpgAF/NpgAR2. The 1116 bp product was digested with *SnaB*I/*Not*I and ligated into vector pPIC3.5 K opened with *SnaB*I/*Not*I to yield the expression vector pPIC3.5 K-NpgA. After transforming into *E. coli* TOP 10, PCR verification by primers 5AOX1/3AOX1 and sequencing, the correct plasmid was then linearized with *Sal*I and transformed by electroporation into wild-type *P. pastoris* strain GS115 and the positive transformant GS115-NpgA was selected with histidine self-synthesis ability. The MSAS encoding gene *atX* was first amplified from the plasmid pESC-ATX recevied from Prof. Fujii [18]. The primers AtxF/AtxR were used and a DNA fragment of 5437 bp was harvested. It was then digested by *EcoR*I/*Not*I and ligated into *EcoR*I/*Not*I sites of the opened vector pPICZ B to yield the expression plasmid pPICZ B-ATX. The plasmid was transformed into *E. coli* Top 10, PCR verification by primers BstPF/AtxR and tested by sequencing. The correct plasmid was then amplified and digested by *Pme*I and then transformed by electroporation into *P. pastoris* GS115 and GS115-NpgA to form recombinant strains GS115-ATX and GS115-NpgA-ATX.

### Strain GS115-3.5 K, GS115-3.5 K-CT-SKL, GS115- NpgA-CT-SKL, GS115-NpgA-CT and GS115-NpgA-SKL-CT-SKL

The commerial vector pPIC3.5 K acting as a control was transformed *P. pastoris* GS115 to gain strain GS115-3.5 K. A *pksCT* gene coding for citrinin synthase (PksCT) was first identified by reverse transcription to harvest cDNA [21]. Primer pairs CTAF/CTAR, CTBF/CTBR and CTCF/CTCR were used to clone three fragments of *pksCT* and then they were digested and ligated into pPICZ B successively to produce a plasmid pPICZ B-CT-SKL having the complete *pksCT* gene with a SKL sequence for peroxisomal targeting. The plasmid was transformed into *E. coli* Top 10, PCR verification by primers CTAF/CTCR and tested by sequencing. The correct plasmid was then amplified and digested by *Pme*I and then transformed by electroporation into *P. pastoris* GS115-NpgA and GS115-3.5 K to form GS115-NpgA-CT-SKL and GS115-3.5 K-CT-SKL, respectively. Then, primers Citrinin-F2/ Citrinin-R2 were used for cloning a *pksCT* fragment from pPICZ B-CT-SKL and it was then digested by *Cla*I/*Not*I and ligated into pPICZ B-CT-SKL opened with *Cla*I/*Not*I to form pPICZ B-CT that removing the SKL sequence. The pPICZ B-CT transformed GS115-NpgA to get strain GS115-NpgA-CT. A pPIC3.5 K-NpgA-SKL plasmid was then constructed similar to pPIC3.5 K-NpgA, which only differed that involving a SKL sequence by primers NpgAF/NpgASR1 followed by NpgAF/NpgASR2 to direct NpgA into peroxisome. The pPIC3.5 K-NpgA-SKL transformed GS115 to generate GS115-NpgA-SKL, which was then transformed by pPICZ B-CT-SKL and produced GS115-NpgA-SKL-CT-SKL.

### Strain GS115-NpgA-HIS_6_

Genomic DNA of *A. nidulans* A1149 was first extracted. Primers NpgA-HIS_6_-F/NpgA-HIS_6_-R were designed and used for cloning *npgA* coding for PPTase from *A. nidulans* A1149 by involving His-tag and a PCR product of 1106 bp was obtained. The product was digested with *Sac*II/*Not*I and ligated into the same sites of the digested vector pPICZ B to yield the expression vector pPICZ B-NpgA-HIS_6_. After transforming into *E. coli* TOP 10, PCR verification by primers 5AOX1/3AOX1 and sequencing, the correct plasmid was then linearized with *Pme*I and transformed by electroporation into wild-type auxotrophic *his4* strain *P. pastoris* GS115 to produce the recombinant strain GS115-NpgA-HIS_6_ with zeocin resistence.

### Strains *E. coli* BL21-ACP and *E. coli* BL21-ACPm

The UMA method was applied to identify the ACP domain of the citrinin synthase (PksCT) from *M. purpureus*[22]. Then primers AcpF/AcpR with *Nhe*I/*Xho*I sites were designed based on the obtained DNA sequence. An ACP encoding DNA fragment of 534 bp was then cloned using primers AcpF/AcpR and cDNA from *M. purpureus*. The product was digested with *Nhe*I/*Xho*I and ligated into *Nhe*I/*Xho*I sites of the digested vector pET28a to yield the expression vector pET28a-ACP. The plasmid was transformed into *E. coli* Top 10, PCR verification by primers 5AOX1/3AOX1 and tested by sequencing. The correct plasmid was then transformed into *E. coli* BL21 to yield recombinant strain BL21-ACP for protein expression.

As PPtase carries coenzyme A to the hydroxyl of conserved Ser residue in ACP to make it work, mutation at serine would inactivate it. Thus DNA fragment of ACPm were then constructed by over-lap PCR with primers mutant5/mutant3 to change conserved serine at site 56 to alanine (Figure 5A). The strain BL21-ACPm was then constructed following the same procedure of strain pET28a-ACP.

### Medium and agent

MGY medium: glycerol 10 g/L, YNB (Sigma) 6.7 g/L, sterilized at 121°C for 20 min. Biotin was added to 0.4 mg/L after cooling.

MM medium: YNB (Sigma) 13.4 g/L, sterilized at 121°C for 20 min. Biotin was added to 0.4 mg/L and methanol was added to 0.5% (v/v) after cooling.

BSM medium: 85% (w/v) H_3_PO_4_ 13 mL/L, KOH 10.6 g/L, CaSO_4_ 0.82 g/L, K_2_SO_4_ 18.2 g/L, MgSO_4_ · 7H_2_O 14.9 g/L, glycerol 40 g/L, (NH_4_)_2_SO_4_ 13.2 g/L, Antifoam 204 (Sigma) 0.33 mL/L, sterilized at 121°C for 30 min. PTM1 solution was then added with 4.4 mL/L after cooling.

PTM1 solution: CuSO_4_ · 5H_2_O 6.0 g/L, NaI 0.08 g/L, MnSO_4_ · H_2_O 3.0 g/L, Na_2_MoO_4_ · 2H_2_O 0.2 g/L, HBO_3_ 0.02 g/L, CoCl_2_ · 6H_2_O 0.914 g/L, ZnCl_2_ 20.0 g/L, FeSO_4_ · 7H_2_O 65.0 g/L, biotin 0.2 g/L, H_2_SO_4_ 5.0 mL/L, sterilized by filtration with 0.22 μm membrane filter and stored at 4°C away from light.

Glycerol feeding medium: glycerol 50% (w/v), sterilized at 121°C for 30 min. PTM1 solution was then added with 12 mL/L after cooling.

Methanol feeding medium: methanol (100%) with 12 mL/L PTM1.

SDS-PAGE electrophoresis buffer: 1 g SDS, 3.03 g Tris, 14.4 g Glycine, dissolved in 1 L ultrapure water.

Coomassie brilliant blue R250 solution: 1.0 g Coomassie brilliant blue R250, 100 mL acetic acid, 300 mL ethanol, dissolved in 1 L ultrapure water.

Electrophoretic destain solution: 32 mL acetic acid, 100 mL ethanol, ultrapure water added to 1 L.

SDS-PAGE loading buffer (5×): 1 g SDS, 2.5 mL 1 M Tris–HCl (pH 6.8), 5 mL glycerol, 0.05 g Coomassie brilliant blue, ultrapure water added to 10 mL.

Nickel-affinity chromatography binding buffer: 10 mmol/L NaH_2_PO_4_, 10 mmol/L Na_2_HPO_4_, 0.5 mol/L NaCl, 20 mmol/L imidazole, pH 7.4.

Nickel-affinity chromatography elution buffer: 10 mmol/L NaH_2_PO_4_, 10 mmol/L Na_2_HPO_4_, 0.5 mol/L NaCl, 500 mmol/L imidazole, pH 7.4.

Fluorescent labeling reaction buffer: 137 mM NaCl, 10 mM PBS (pH 7.4), 50 mM MgCl_2_.

CoA reaction buffer:75 mM Tris–HCl (pH 8.8), 10 mM MgCl_2_, 25 mM DTT.

20 mM Tris dialysis buffer: 20 mM Tris–HCl (pH 7.5), 150 mM NaCl.

### Culture conditions

For GS115-NpgA-HIS_6_, a singal colony was inoculated into a 250 mL shake flask containing 25 mL YPD medium and cultivated at 30°C and 200 rpm for about 16 hours. Afterwards, the broth was transferred into 25 mL MGY medium to make the OD_600_ about 0.03. The liquid was centrifugated at 3000 *g* for 5 min, washed with MM medium twice and suspended with MM medium to be OD_600_ = 1.0. Then 50 mL liquid was then transferred into 500 mL baffled shake flask, covered two layers of gauze and cultivated at 30°C and 200 rpm. Methanol was fed every 24 hours to keep its concentration around 0.5% to induce protein expression.

For BL21-ACP and BL21-ACPm, a singal colony was inoculated into a 250 mL shake flask containing 25 mL LB medium with 50 μg/mL kanamycin and incubated at 37°C and 200 rpm for 12 hours. Then it was inoculated into 25 mL LB medium with 50 μg/mL kanamycin in 250 mL shake flask by 1% (v/v) and cultivated at 37°C and 200 rpm until OD_600_ = 0.6. Afterwards, 0.5 mM IPTG was added to induce protein expression for 5 hours. The broth was centrifugated at 8000 *g* for 3 min, washed with binding buffer and then stored at −20°C for forward analysis.

The shake flask cultures of GS115-ATX, GS115-NpgA-ATX, GS115- NpgA-CT-SKL, GS115-3.5 K-CT-SKL, GS115-NpgA-CT and GS115-NpgA-SKL-CT-SKL were as same as that of GS115-NpgA-HIS_6_. For 5-L bioreactor, 300 mL seeds (OD_600_ = 6.0) cultivated with MGY medium were collected and inoculated into a 5-L stirred-tank bioreactor (Shanghai Guoqiang Bioengineering Equipment Co., Ltd.) containing 3 L BSM medium. The impeller equipped was double layer six-blade Rushton disc turbine (RDT, 6.8 cm i.d.). The lower impeller was 2.5 cm above the reactor bottom, and the vertical distance between two impellers was 7.2 cm. Dissolved oxygen (DO) was measured using a polarographic probe calibrated to 100% saturation for aeration of 1 vvm at agitation of 600 rpm and tank inside pressure of 0.02 Mpa. The broth pH was controlled at 5.0 by NH_4_OH. The temperature was kept at 30°C and the DO was controlled over 30% by adjusting agitation (not higher than 800 rpm) and aeration (mixed gas of oxygen and air if needed). When glycerol used up and DO repidly increased, glycerol feeding medium was limitedly fed by 8 mL/L/h for 2 hours. The feeding rate may accelerate but keep DO not lower than 30% until the wet cell weight (WCW) reached 250 g/L. After glycerol was exhausted, methanol feeding medium were fed by 4 mL/h/L for 2 hours and then slowly increased to 12 mL/h/L after 6 more hours and keep this rate until the end.

### Protein expression and purification

To prepare PPtase, 30 OD_600_ units of GS115-NpgA-HIS_6_ cells were harvested by centrifugation at 6,000 *g* for 3 min, washed twice with ice-cold 50 mM binding buffer (pH 7.4), and then frozen at −20°C. Cells were thawed and resuspended in 1 mL binding buffer (pH 7.4). It were mixed with 1.8 g glass beads (Biospec Products, Bartlesville, OK) in a 2.0-mL screw-cap tube followed by disruption with a bead disrupter (Mini-BeadBeater-8; Biospec Products) for 8 cycles (1 min vibrating and 1 min resting in ice for each cycle). The lysate was centrifuged at 12,000 *g* for 30 min and the solid was discarded. It was then filtrated by 0.22 μm membrane and loading to the column that balanced well with binding buffer by 0.5 mL/min. Afterwards, the column was washed with binding buffer of 10 column volumes to discard other proteins, followed by elution buffer of 5 column volumes to collect the proteins. The eluent was dialyzed by 20 mM Tris–HCl (pH 7.5) twice and concentrated to 4 mg/mL by ultrafiltration, and then used for *in vitro* experiment.

Cells of BL21-ACP and BL21-ACPm were centrifugated and resuspended with 20 mM Tris–HCl (pH 7.5). The proteins were then extracted by ultrasonic disruption (300 w, 3 s on, 4 s off, 15 min) and collected by centrifugation at 12000 *g* and 4°C for 20–30 min (supernatant).

Eluted samples were analyzed on a Tris–Gly SDS-PAGE gel (Invitrogen) and stained with Coomassie brilliant blue R250 solution.

### Fluorescence labeling of CoA and bioactivity analysis of PPTase

Preparation of Alexa Fluor 647-CoA: Dissolve 0.02 mg Alexa Fluor 647-C2maleimide in 20 μL 50% DMSO. It was then mixed with 1.0 mg CoA (0.53 μM) and 30% DMSO (v/v), fed with fluorescent labeling reaction buffer up to 245 μL, and reacted in dark for 1 hour. The Alexa Fluor 647-CoA was then purified referring to previous study [25].

Preparation of Bodipy FL-CoA: Dissolve 1 mg bodipy FL N-(2-aminoethyl)maleimide 40 μL DMSO. It was then mixed with 0.8 mg CoA and 10% DMSO (v/v), fed with fluorescent labeling reaction buffer up to 2 mL and reacted in dark for 85 min. The liquor was then extracted with ethyl acetate for three times and the aqueous phase with Bodipy FL-CoA was collected.

The 25 μL reaction system consisted of 9.5 μM Alexa Fluor 647-CoA (or 2 μL Bodipy-CoA), 0.16 μg/μL purified His6-NpgA (PPtase), 5 μL (200 μg) protein supernant of BL21-ACP(or BL21-ACPm) in CoA reaction buffer. After incubated at room temperature for 10 min, loading buffer was added to terminate the reaction and used for SDS-PAGE after heated at 100°C for 2 min.

The KODAK In-Vivo multispectral imaging system was used for the gel analysis by setting different wave length of exciting light. After analysis, the gel was photoed under white light after stained by Coomassie brilliant blue R250 solution and destained in electrophoretic destain solution.

### Extraction and identification of 6-MSA and citrinin intermediate

For 6-MSA, 10 mL culture broth was centrifugated at 12000 *g* for 3 min. The supernatant was fully extracted with 20 mL ethyl acetate, and the organic phase was distilled under reduced pressure and dissolved in 10 mL methanol. It was used for HPLC analysis after filtration and dilution. The cells was resuspended in 1 mL methanol and disrupted by bead disrupter just as described before. The mixture was then centrifugated at 12000 *g* for 3 min and the obtained supernant was filtrated and diluted before HPLC analysis.

The sample was analyzed by a HPLC system of Agilent 1100 with a C18 column (Kromasil™, Sweden, 250 mm × 4.6 mm × 5 μm, 100 Å-spherical silica). It was eluted with a gradiant strategy that 90% (v/v) A (water + 1% AcOH) and 10% (v/v) B (CH3CN + 1% AcOH) to 20% (v/v) A and 80% (v/v) B in 30 min, then to 100% (v/v) B in 2 min and kept for 5 min. Afterwards, it was then gradiantly changed to 90% (v/v) A and 10% (v/v) B in 3 min. The elution conditions were set as that flow rate of 1.0 mL/min, UV absorbency of 306 nm and operating temperature of 30°C. The compound was further confirmed by mass spectrometry (EI-MS, Agilent G2577A), and also nuclear magnetic resonance (NMR, BRUKER-400) with freeze-dried sample dissolved in deuterated DMSO for ^1^HNMR analysis. For quantifying the production of 6-MSA, the standard 6-MSA (Shanghai Bepharm Co. Ltd) was used for standard curve preparation by the same HPLC procedure. The extracted 6-MSA samples from fermentation broth were then diluted properly for HPLC analysis and the 6-MSA production in fermentation broth was then calculated. The compound was also confirmed by EI-MS and ^1^HNMR analysis.

Extraction of citrinin intermediate was as same as 6-MSA. The HPLC system used was also the same. It was eluted with a gradiant strategy that 90% (v/v) A (water + 1% AcOH) and 10% (v/v) B (CH3CN + 1% AcOH) to 30% (v/v) A and 70% (v/v) B in 25 min, then immediately change to 100% (v/v) B and kept for 5 min. The elution conditions were set as that flow rate of 1.0 mL/min, UV absorbency of wide wavelength range and operating temperature of 30°C.

### Transcription and western blot analysis

Total RNAs were prepared by RiboPure™-Yeast Kit (Ambion) following its standard protocol and were subjected to DNase I treatment to exclude the genomic DNA contaminant. Reverse transcription was performed following ReverTra Ace transcription kit (Toyobo). For western blot, samples were first prepared as same as that described in the Section of Protein expression and purification. Total proteins were separated on polyacrylamide gels under denaturing conditions [31] and then transferred onto a polyvinylidene difluoride (PVDF) membrane using the electrophoretic transfer method with rabbit anti-HA antibody (Tiangen) as the primary antibody and peroxidase-conjugated goat anti-rabbit immunoglobulin G (Jackson ImmunoResearch) as the secondary antibody.

## Competing interests

The authors declare that they have no competing interests.

## Authors’ contributions

LG and MC designed the experiments, LG conducted most of the experiments. LG, WS and SX performed bioreactor fermentations. LG and MC analyzed the results. MC wrote the manuscript. XZ and YZ reviewed and revised the manuscript. All authors read and approved the final manuscript.

## Supplementary Material

Additional file 1: Figure S1 Time profiles of *P. pastoris* GS115 in 5-L stirred-tank bioreactor fermentation with 6-MSA feeding. The first arrow (24 h) indicated the starting point of glycerol feeding; The second arrow (32 h) indicated the time point of 6-MSA feeding. Click here for file
