# Supplementary material for: Engineered fungal polyketide biosynthesis in Pichia pastoris: a potential excellent host for polyketide production
Source: Microb Cell Fact. 2013 Sep 8;12:77. doi: 10.1186/1475-2859-12-77 (PMC3847973; doi:10.1186/1475-2859-12-77)
Supplement: Additional file 1: Figure S1 — Time profiles of P. pastoris GS115 in 5-L stirred-tank bioreactor fermentation with 6-MSA feeding. The first arrow (24 h) indicated the starting point of glycerol feeding; The second arrow (32 h) indicated the time point of 6-MSA feeding. [file 1475-2859-12-77-S1.pdf]

**Supplementary Data**

# Engineered fungal polyketide biosynthesis in *Pichia pastoris*: a potential excellent host for polyketide production

Limei Gao[1], Menghao Cai[1,*], Wei Shen[1], Siwei Xiao[1], Xiangshan Zhou[1], Yuanxing Zhang[1]

**Affiliation and address**

[1] State Key Laboratory of Bioreactor Engineering, East China University of Science and Technology, Shanghai 200237, China

***Corresponding author**

Tel.: +86-21-64253065; Fax: +86-21-64253025.

E-mail: cmh022199@ecust.edu.cn (Menghao Cai)

**First author**

E-mail: gaolimei2006@163.com (Limei Gao)

**Other co-authors**

E-mail: 19881004shen@163.com (Wei Shen), xsw736817@126.com (Siwei Xiao), yxzhang@ecust.edu.cn (Yuanxing Zhang), xszhou@ecust.edu.cn (Xiangshan Zhou)

For 5-L bioreactor, 330 mL seeds ($OD_{600}$ = 6.0) cultivated with MGY medium were collected and inoculated into a 5-L stirred-tank bioreactor (Shanghai Guoqiang Bioengineering Equipment Co., Ltd.) containing 3 L BSM medium. The impeller equipped was double layer six-blade Rushton disc turbine (RDT, 6.8 cm i.d.). The lower impeller was 2.5 cm above the reactor bottom, and the vertical distance between two impellers was 7.2 cm. Dissolved oxygen (DO) was measured using a polarographic probe calibrated to 100% saturation for aeration of 1 vvm at agitation of 600 rpm and tank inside pressure of 0.02 Mpa. The broth pH was controlled at 5.0 by $NH_4OH$. The temperature was kept at 30ºC and the DO was controlled over 30% by adjusting agitation (not higher than 800 rpm) and aeration (mixed gas of oxygen and air if needed). When glycerol used up and DO repidly increased, glycerol feeding medium was limitedly fed by 8 mL/L/h for 2 hours. The feeding rate may accelerate but keep DO not lower than 30% until the wet cell weight (WCW) reached 235 g/L at 32.3 h. Then, 6-MSA with final concentration of 2.05 g/L was fed and the glycerol feeding was kept at 8 mL/L/h until 39.3 h.

As shown in Suppl. Fig. 1, immediately after 6-MSA addition, the DO sharply inceased to over 90% and remained at hight levels until the end. That indicated that cell metabolism was severely damaged. Cell growth was also highly repressed and the WCW gradually decreased after 6-MSA feeding. Methylene blue staining of the cells showed a great amount of blue dead cells. The results proved that 6-MSA was harmful to *P. pastoris*.

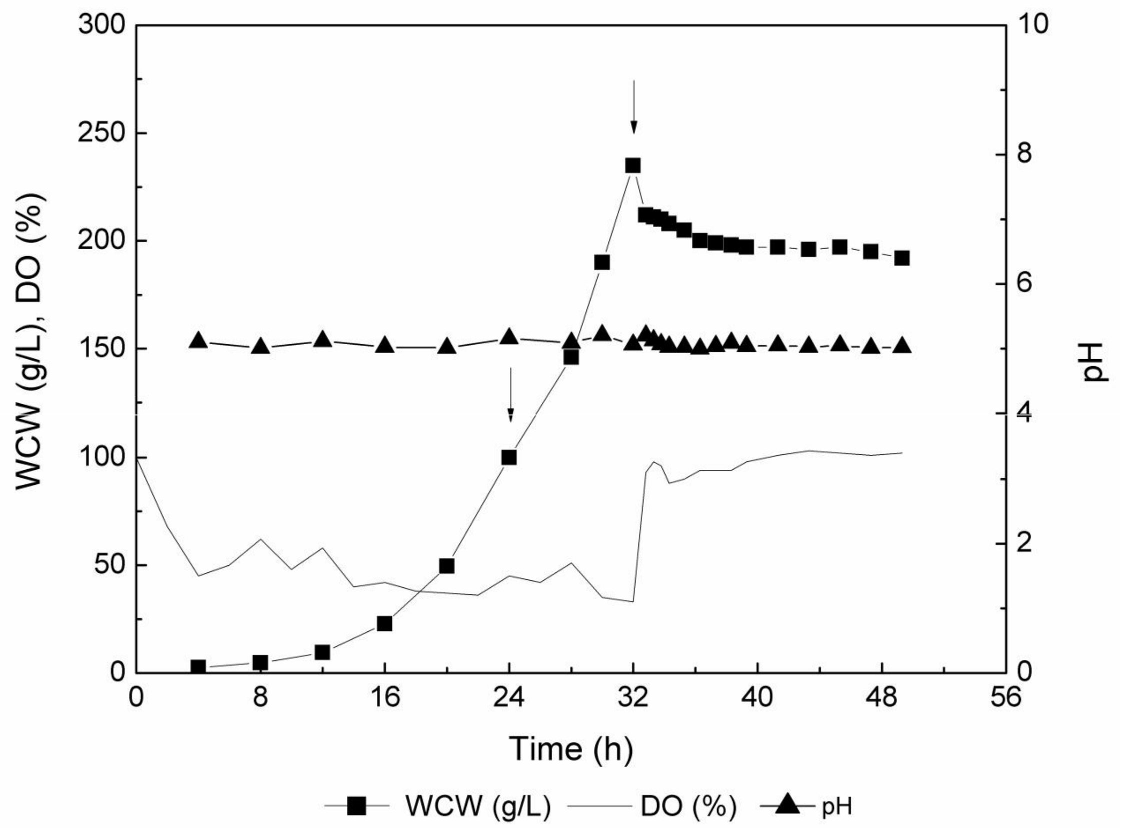


**Suppl. Fig. 1 Time profiles of *P. pastoris* GS115 in 5-L stirred-tank bioreactor fermentation with 6-MSA feeding.** The first arrow (24 h) indicated the starting point of glycerol feeding; The second arrow (32 h) indicated the time point of 6-MSA feeding.
